# Spearmint R2R3‐MYB transcription factor MsMYB negatively regulates monoterpene production and suppresses the expression of geranyl diphosphate synthase large subunit (*MsGPPS*.*LSU*)

**DOI:** 10.1111/pbi.12701

**Published:** 2017-03-18

**Authors:** Vaishnavi Amarr Reddy, Qian Wang, Niha Dhar, Nadimuthu Kumar, Prasanna Nori Venkatesh, Chakravarthy Rajan, Deepa Panicker, Vishweshwaran Sridhar, Hui‐Zhu Mao, Rajani Sarojam

**Affiliations:** ^1^ Temasek Life Sciences Laboratory National University of Singapore Singapore Singapore; ^2^ Department of Biological Sciences National University of Singapore Singapore Singapore; ^3^ Present address: College of Biological and Environmental Sciences Zhejiang Wanli University Ningbo Zhejiang 315100 China; ^4^ Present address: Singapore Centre on Environmental Life Sciences Engineering Nanyang Technological University Singapore 637551 Singapore

**Keywords:** transcription factor, R2R3‐MYB, secondary metabolism, spearmint, GPPS, terpene

## Abstract

Many aromatic plants, such as spearmint, produce valuable essential oils in specialized structures called peltate glandular trichomes (PGTs). Understanding the regulatory mechanisms behind the production of these important secondary metabolites will help design new approaches to engineer them. Here, we identified a PGT‐specific R2R3‐MYB gene, *MsMYB,* from comparative RNA‐Seq data of spearmint and functionally characterized it. Analysis of *MsMYB*‐RNAi transgenic lines showed increased levels of monoterpenes, and *MsMYB‐*overexpressing lines exhibited decreased levels of monoterpenes. These results suggest that MsMYB is a novel negative regulator of monoterpene biosynthesis. Ectopic expression of *MsMYB,* in sweet basil and tobacco, perturbed sesquiterpene‐ and diterpene‐derived metabolite production. In addition, we found that MsMYB binds to *cis*‐elements of *MsGPPS*.*LSU* and suppresses its expression. Phylogenetic analysis placed MsMYB in subgroup 7 of R2R3‐MYBs whose members govern phenylpropanoid pathway and are regulated by miR858. Analysis of transgenic lines showed that *MsMYB* is more specific to terpene biosynthesis as it did not affect metabolites derived from phenylpropanoid pathway. Further, our results indicate that *MsMYB* is probably not regulated by miR858, like other members of subgroup 7.

## Introduction

Plants produce an overwhelming variety of specialized metabolites essential for ecological interactions, among which terpenes form the largest and structurally diverse class of natural products. They are involved in mediating plant defence responses and plant pollinator attraction and facilitate plant–environment interactions (Bhargava *et al*., 2013; Pichersky and Gershenzon, 2002; Singh and Sharma, 2015). Apart from imparting ecological benefits to plants, terpenoids are of great economic importance to humans as well, as they are widely used in flavours, fragrances, cosmetics, pharmaceuticals, agricultural industries and chemical industries (Bouvier *et al*., 2005). Given the commercial and ecological importance of terpenoids, strategies to metabolically engineer them are of considerable interest. Monoterpenes form the C_10_ class of terpenoids and are generally colourless, lipophilic and volatile. They are responsible for the characteristic aromas and flavours of essential oils, floral scents and resin of aromatic plants (Loza‐Tavera, 1999). The essential oil of spearmint (*Mentha spicata*) mainly consists of two monoterpenes, limonene and carvone, which are extensively exploited for their biological properties (Ringer *et al*., 2005). In spearmint, the essential oils are produced and stored in specialized structures called peltate glandular trichomes (Figure S1a). These so‐called green biofactories are widely found on aerial surfaces of many aromatic plants, and they actively produce and store large quantity of volatile metabolites (Champagne and Boutry, 2013; Lange and Turner, 2013; Turner *et al*., 2000).

Terpene biosynthesis pathway has been well studied in plants. The universal C_5_ building blocks for all types of terpenes, isopentenyl diphosphate (IPP) and its isomer, dimethylallyl diphosphate (DMAPP), are synthesized either by the mevalonate (MVA) pathway in the cytosol or by the 2‐C‐methyl‐D‐erythritol 4‐phosphate (MEP) pathway in plastids (Vranova *et al*., 2013). The plastidial MEP pathway is largely responsible for producing C_5_ precursors for monoterpenes and diterpenes production, whereas the cytosolic MVA pathway generates C_5_ precursors for sesquiterpene and triterpene production (Dubey *et al*., 2003). However, few studies indicate that, under certain conditions, an exchange of precursor metabolites can occur between the cytosolic MVA and plastidial MEP pathways (Hemmerlin *et al*., 2012; Vranova *et al*., 2013). IPP and DMAPP undergo successive condensation reactions, catalysed by a class of enzymes called prenyltransferases, to form intermediates geranyl diphosphate (GPP; C_10_), farnesyl diphosphate (FPP; C_15_) and geranylgeranyl diphosphate (GGPP; C_20_). These terpene diphosphates form the immediate precursors of monoterpenes, sesquiterpenes and diterpenes, respectively (Kellogg and Poulter, 1997; Liang, 2009; Liang *et al*., 2002; Ogura and Koyama, 1998). Prenyltransferases are key to terpene production as they control the IPP flux into various branches of the terpene family (Liang *et al*., 2002; Oldfield and Lin, 2012).

Geranyl diphosphate synthase (GPPS) is the prenyltransferase enzyme that is mainly responsible for the production of monoterpene precursor GPP in the plastids. It catalyses a single condensation of terpene precursors DMAPP and IPP, to form GPP. Studies in few species of plants revealed that GPPS can function as a homodimer or heterodimer (Nagegowda, 2010). Heteromeric GPPSs have been characterized so far only in angiosperms *Mentha piperita, Antirrhinum majus, Clarkia breweri* and *Humulus lupulus*. All these plants produce large amounts of monoterpenes in specialized organs such as PGTs and flower petals (Burke *et al*., 1999; Tholl *et al*., 2004; Wang and Dixon, 2009). Heteromeric GPPS in mint consist of a large subunit (LSU) and a small subunit (SSU); both LSU and SSU are catalytically inactive alone (Burke and Croteau, 2002; Burke *et al*., 1999; Croteau *et al*., 2005). Interaction between the two subunits results in the formation of an active GPPS. Structural studies show that the LSU acts as the catalytic unit, whereas the SSU serves as the regulatory unit (Chang *et al*., 2010).

In the case of spearmint, the monoterpene biosynthetic pathway is well characterized (Champagne and Boutry, 2013; Croteau *et al*., 1991; Lange *et al*., 2011; Munoz‐Bertomeu *et al*., 2008). Strategies to increase the yield of peppermint oil by manipulating genes that code for structural pathway enzymes have been reported (Diemer *et al*., 2001; Mahmoud *et al*., 2004). However, the developmental regulation of this secondary metabolite pathway still remains elusive. Transcription factors (TFs) can activate or repress multiple genes in a metabolic pathway; hence, they are ideal targets for pathway engineering (Grotewold, 2008; Iwase *et al*., 2009). Only one TF *MsYABBY5* has been reported from spearmint that regulates monoterpene production (Wang *et al*., 2016) but several TFs have been identified from other plants like *Artemisia*, cotton, *Taxus*, rubber and rice that control terpene biosynthesis (Chen *et al*., 2012; Li *et al*., 2013; Miyamoto *et al*., 2014; Shen *et al*., 2016; Xu *et al*., 2004; Zhang *et al*., 2012).

The R2R3‐MYBs represent one of the largest families of plant TFs (Kranz *et al*., 1998; Stracke *et al*., 2001) and can function as activators or repressors of genes (Adato *et al*., 2009; Legay *et al*., 2007). Many R2R3‐MYBs have been characterized as transcriptional activators of flavonoid, phenylpropanoid and glucosinolate pathway genes in plants (Dubos *et al*., 2010; Zhao and Dixon, 2011; Zhong *et al*., 2010). R2R3‐MYBs belonging to subgroup 4 are known to negatively regulate monolignol and flavonoid biosynthetic pathways (Fornalé *et al*., 2010; Jin *et al*., 2000; Legay *et al*., 2007; Preston *et al*., 2004; Tamagnone *et al*., 1998). Further, a single R2R3‐MYB gene was also found to act as both repressor and activator of genes (Bhargava *et al*., 2010). Redundancy, dual function and opposite action as activators and repressors on common target genes by R2R3‐MYBs help fine tune the regulation of plant secondary metabolite pathways. Despite several studies on R2R3 MYB‐mediated regulation of plant phenylpropanoid pathway, knowledge of its role in terpene secondary metabolism is lacking (Matus, 2016). Besides TFs, microRNAs also are known regulators of various processes in plant growth and development. However, knowledge of their role in controlling secondary metabolic pathways is limited. microRNAs mainly target TFs to regulate various plant developmental processes (Sunkar *et al*., 2012; Wu *et al*., 2013; Zhang, 2015; Zhang and Wang, 2015). In plants, MYB TFs are potential targets for miR858 (Addo‐Quaye *et al*., 2008; Guan *et al*., 2014; Jeong *et al*., 2013; Jia *et al*., 2015; Xia *et al*., 2012). Further, many studies have shown that miR858 can target R2R3‐MYBs in apple, grapes, salvia and Arabidopsis (Li and Lu, 2014; Rock, 2013; Sharma *et al*., 2016; Xia *et al*., 2012). Presently, miR858 is considered as a potential regulator of plant phenylpropanoid metabolite pathway through its action on R2R3‐MYBs (Fahlgren *et al*., 2007).

In this study, we identified a R2R3‐MYB transcript which is preferentially expressed in spearmint PGTs from the comparative transcriptome data (Jin *et al*., 2014) of spearmint leaf, leaf stripped of PGT (L‐PGT) and PGT. Phylogenetic analysis assigned MsMYB to subgroup 7 of R2R3‐MYBs of Arabidopsis. Members of subgroup 7 are closely related R2R3‐MYBs which control flavonoid accumulation in different parts of the Arabidopsis seedling, and are regulated by miR858 (Fahlgren *et al*., 2007; Sharma *et al*., 2016; Yang *et al*., 2012). Apart from Arabidopsis, several other R2R3‐MYBs from different plants, similar to the members of subgroup 7, are also involved in flavonoid pathway (Adato *et al*., 2009). We functionally characterized the role of this PGT‐specific R2R3‐MYB and found it to be a negative regulator of monoterpene production in spearmint. Suppression of *MsMYB* expression*,* by RNAi approach, led to increase in monoterpene levels and its overexpression led to decrease in monoterpene levels in transgenic lines. This is the first report of regulation of monoterpene production by a R2R3‐MYB. Ectopic expression of *MsMYB* in sweet basil (*Ocimum basilicum*), an aromatic plant similar to spearmint, and in tobacco (*Nicotiana sylvestris)*, affected the production of terpenes but not of flavonoids. We further found that MsMYB suppresses the expression of geranyl diphosphate synthase large subunit *(MsGPPS.LSU*) gene in spearmint. To investigate whether *MsMYB* is regulated by miR858, similar to other R2R3‐MYBs of subgroup 7, we performed small RNA‐Seq of spearmint PGT and L‐PGT. We found low amounts of miR858 in spearmint PGT but high amounts in L‐PGT. Although *in silico* analysis predicted *MsMYB* as a target for miR858, we were unable to detect its cleaved products. This suggests that the differential expression of miR858 and *MsMYB* in separate tissues prevents *MsMYB* regulation by miR858. Collectively, the above results provide insight into the production of essential oils in a valuable crop plant, such as spearmint, and expand our knowledge of R2R3‐MYB‐mediated regulation of plant secondary metabolism.

## Results

### *MsMYB* shows preferential expression in spearmint PGTs

**Figure 1:**
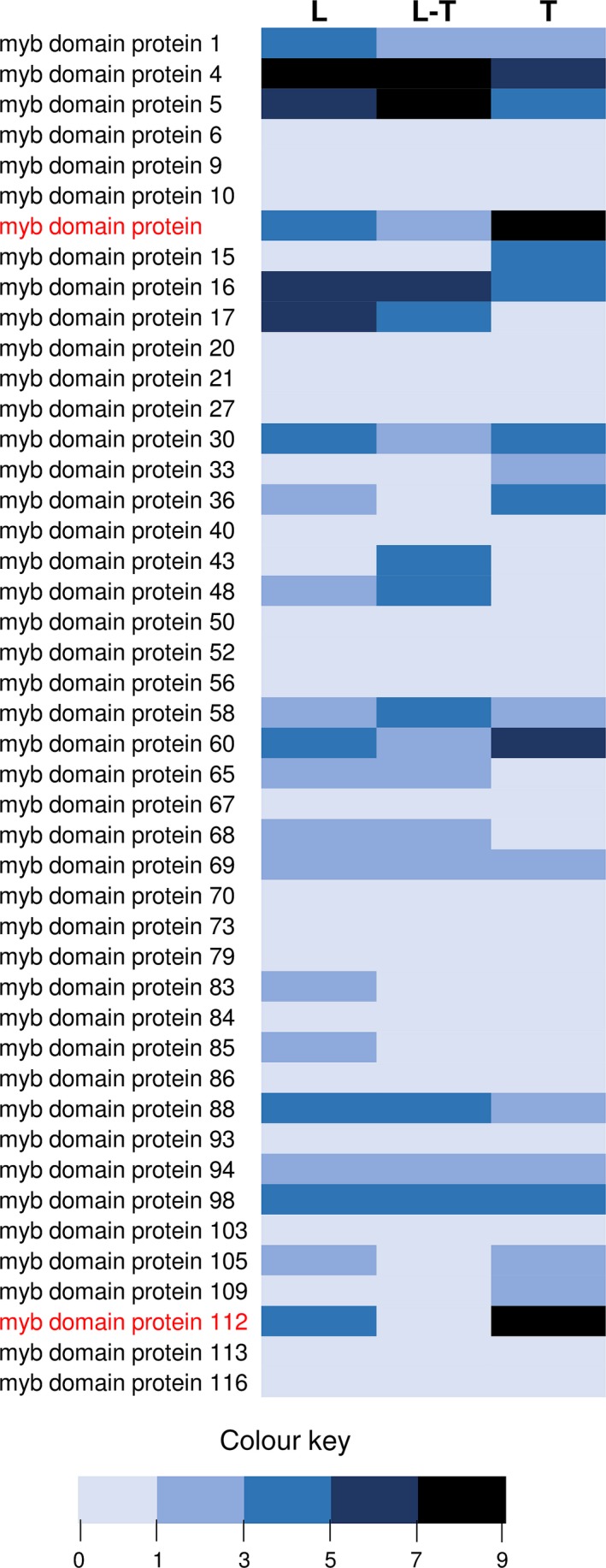
Heat map analysis of spearmint R2R3‐MYBs. Differential expression pattern of 45 annotated R2R3‐MYBs along various tissues [leaf (L), leaf stripped of PGTs (L‐T) and PGTs (T)]. MYB and MYB112 are highlighted in red which show high expression in PGTs. Sequence data and expression values are available in Supplemental Data S1.

**Figure 2:**
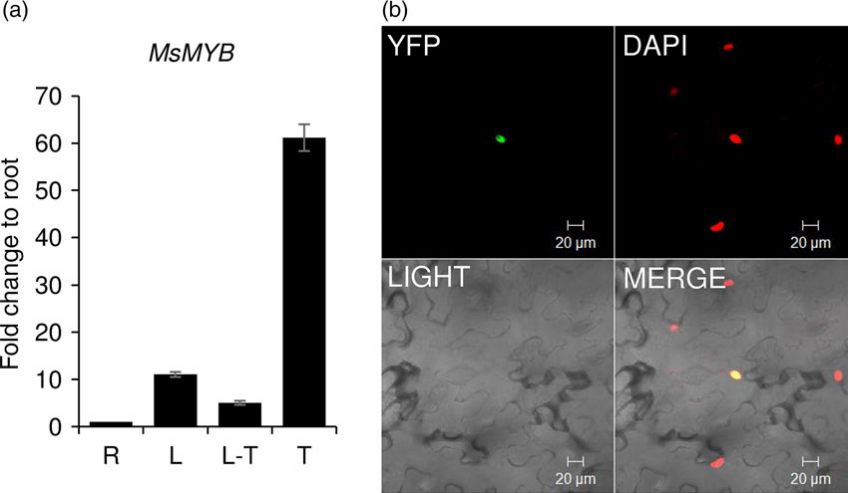
*MsMYB* expression and localization. qRT‐PCR was performed to analyse the expression of *MsMYB* along the various tissues [leaf (L), leaf stripped of PGTs (L‐T), root (R) and PGTs (T)]. (a) Expression levels of *MsMYB* showing preferential expression in PGTs. (b) Nucleus‐specific localization of MsMYB in *N. benthamiana* leaf cells.

**Figure 3:**
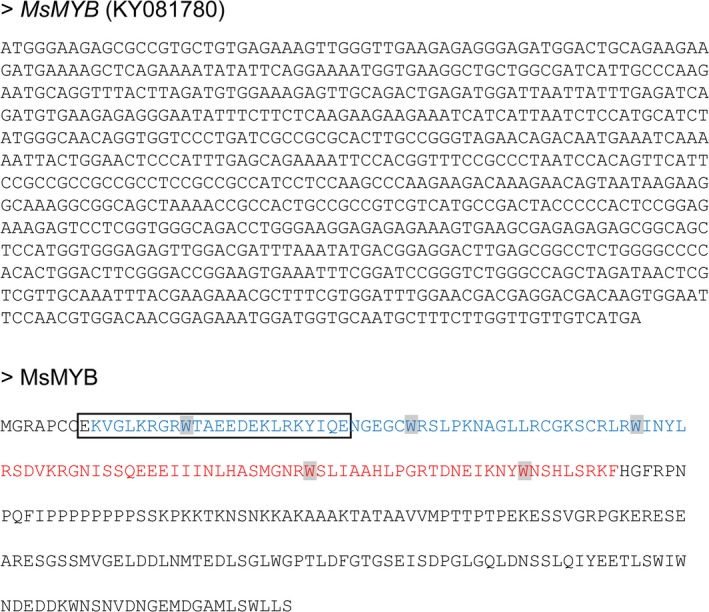
DNA and protein sequence of MsMYB. R2 and R3 repeats are highlighted in blue and red colours, respectively. Five conserved tryptophan residues are highlighted with grey boxes. Nuclear localization signal as predicted by cNLS mapper is shown by a black box.

From the transcriptome data of three tissues of spearmint, namely leaves, PGTs and leaves devoid of PGTs (Jin *et al*., 2014), we annotated 45 R2R3‐MYBs (Figure 1; Supporting Data S1). Of these, only two R2R3‐MYBs, *MsMYB* and *MsMYB112*, showed high expression in PGTs. *MsMYB* was chosen for further characterization. RNA‐Seq expression of *MsMYB* along various tissues was further validated by quantitative RT‐PCR (qRT‐PCR) (Figure 2a). Full‐length open reading frame (ORF) of *MsMYB* was amplified from PGT cDNA and consisted of 813 base pairs (bp) encoding a polypeptide of 271 amino acids (Figure 3). It contained a R2, R3 repeat and five tryptophan residues within the R2 and R3 repeats which together forms a helix‐turn‐helix motif at the N‐terminus. These features are generally conserved among all the R2R3‐MYBs (Dubos *et al*., 2010). MsMYB showed highest sequence similarity (~60%) to *Salvia miltiorrhiza* MYB‐related transcription factor in BLAST analysis. A phylogenetic tree was constructed using the amino acid sequences of known *Arabidopsis thaliana* (At) R2R3‐MYBs (Figure S2). MsMYB fell under subgroup 7 along with AtMYB111, AtMYB11 and AtMYB12 (Dubos *et al*., 2010). All these three Arabidopsis MYBs function as activators of flavonoid pathway (Stracke *et al*., 2007). To examine subcellular localization of MsMYB, the ORF was fused with yellow fluorescent protein (YFP) and expressed under the control of CaMV 35S promoter in *Nicotiana benthamiana* leaves by agroinfiltration. As shown in Figure 2b, the recombinant protein specifically localized to the nucleus.

### *MsMYB* promoter shows trichome specific expression

**Figure 4:**
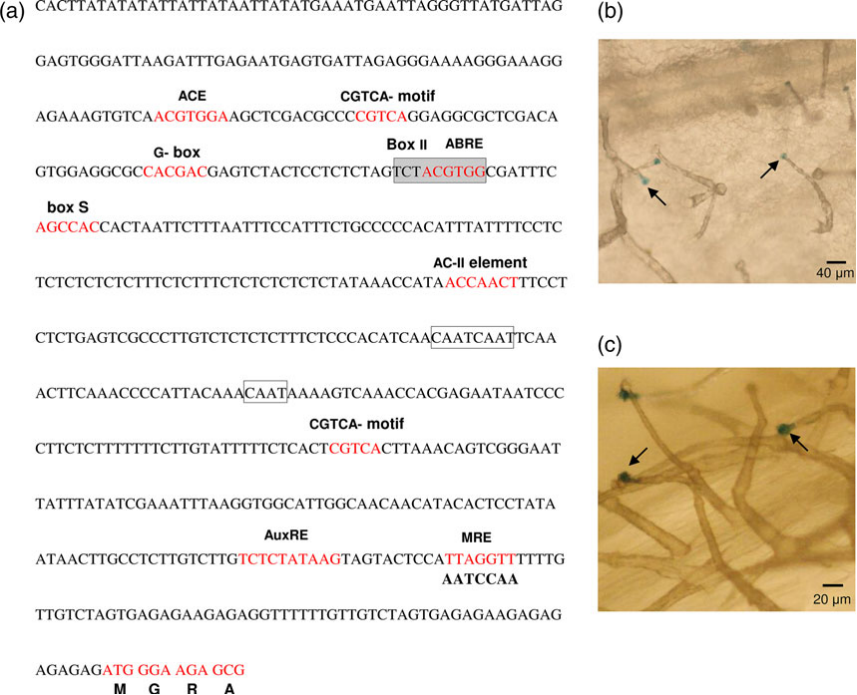
*MsMYB* promoter analysis and expression pattern in tobacco. (a) Regulatory elements present in the promoter region (−615 bp) of *MsMYB*. (b), (c) *N. benthamiana* plants transformed with *MsMYB*_*pro*_ *:GUS* showing trichome specific expression in leaves and stems. Black arrows point GUS stained trichome heads.

To identify the *cis*‐acting regulatory elements in the promoter of *MsMYB,* we cloned a 615‐bp genomic DNA fragment upstream of the translation start site by genome walking. Apart from the common TATA and CAAT box, several other *cis*‐acting regulatory elements were identified (Figure 4a) using PlantCARE tool (http://bioinformatics.psb.ugent.be/webtools/plantcare/html/). Many of these elements were for hormones like ABRE‐motif which is known for abscisic acid responsiveness (Basu *et al*., 2014), AuxRE‐motif which is an auxin‐responsive element (Ulmasov *et al*., 1995), CGTCA‐motif and TGACG‐motif which are known for MeJA responsiveness (Zhou *et al*., 2012). Interestingly an AC‐II element was also found. In general, AC‐rich regions are known to be bound by R2R3‐MYBs (Koschmann *et al*., 2012; Prouse and Campbell, 2012). This suggests that *MsMYB* might be regulated by other R2R3‐MYBs. To check for the expression pattern, the 615‐bp promoter fragment was fused with a β‐glucuronidase (GUS) reporter gene and transformed into *N. benthamiana* plants. Trichome‐specific staining was observed in leaves and stems of tobacco transgenic plants (Figure 4b,c).

### Manipulation of *MsMYB* expression levels affects monoterpene production in spearmint

**Figure 5:**
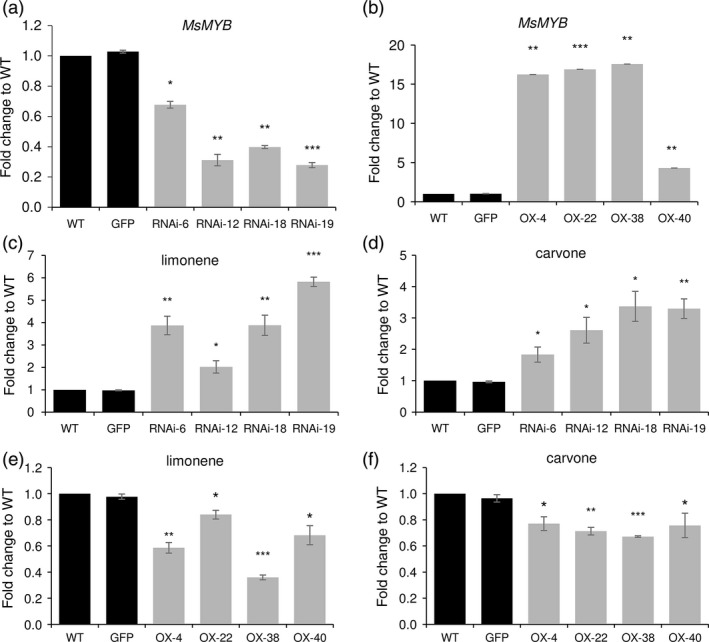
Transcript levels and GC analysis of transgenic plants. Reduced (a) and increased (b) levels of *MsMYB* in transgenic spearmint *MsMYB*‐RNAi and *MsMYB*‐overexpressing lines when compared to WT. (c), (d) Increased levels of limonene and carvone in *MsMYB*‐RNAi lines when compared to WT. (e), (f) Reduced levels of limonene and carvone in *MsMYB*‐overexpressing lines when compared to WT. GFP: GFP‐overexpressing line. Data are indicated as mean ± SE. *P < 0.05; **P < 0.01; ***P < 0.001.

To characterize the function of *MsMYB,* we generated transgenic spearmint lines harbouring RNAi construct and overexpression construct where *MsMYB* was under the control of CaMV 35S promoter. Four independent lines each confirmed by Southern blot were selected for further characterization (Figure S1b,c). qRT‐PCR analysis showed significant reduction in levels of *MsMYB* transcripts in RNAi lines (Figure 5a) and higher levels of *MsMYB* transcripts in overexpression lines when compared to wild type (WT) (Figure 5b). Both overexpression and RNAi transgenic plants appeared phenotypically similar to WT plants. Scanning electron microscopy was performed on these plants to take a closer look at leaf cells and PGTs. No phenotypical changes were observed.

Further, gas chromatography–mass spectrometry (GC‐MS) analysis was performed to evaluate any changes in volatiles production. Major secondary metabolites of spearmint are monoterpenes; limonene and carvone (Figure S3a). GC‐MS analysis revealed significant changes in the amount of these secondary metabolites in transgenic lines. Compared to WT plants, total monoterpene production in *MsMYB*‐RNAi lines was higher; about 2.3‐ to 4.5‐fold increase was observed (Figure 5c,d), whereas in *MsMYB*‐overexpressing lines a decrease of 0.5‐ to 0.7‐fold was seen (Figure 5e,f). The above results indicated that *MsMYB* might be a negative regulator of secondary metabolism in spearmint.

### MsMYB negatively regulates *MsGPPS.LSU*

**Figure 6:**
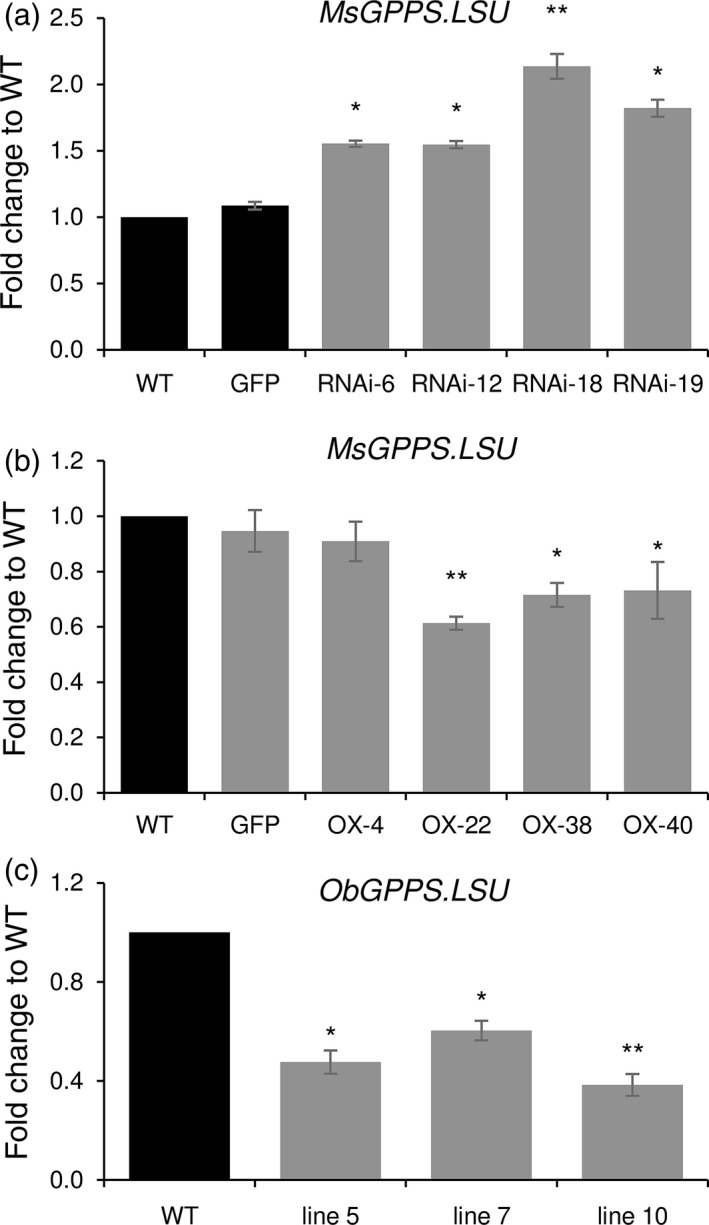
Transcript levels of *GPPS*.*LSU* in transgenic plants. (a) Increased levels of *MsGPPS*.*LSU* in *MsMYB*‐RNAi lines. (b) Decreased levels of *MsGPPS*.*LSU* in *MsMYB*‐OX lines. (c) Decreased levels of *ObGPPS*.*LSU* in sweet basil plants expressing *MsMYB*. Data are indicated as mean ± SE. *P < 0.05; **P < 0.01; ***P < 0.001.

**Figure 7:**
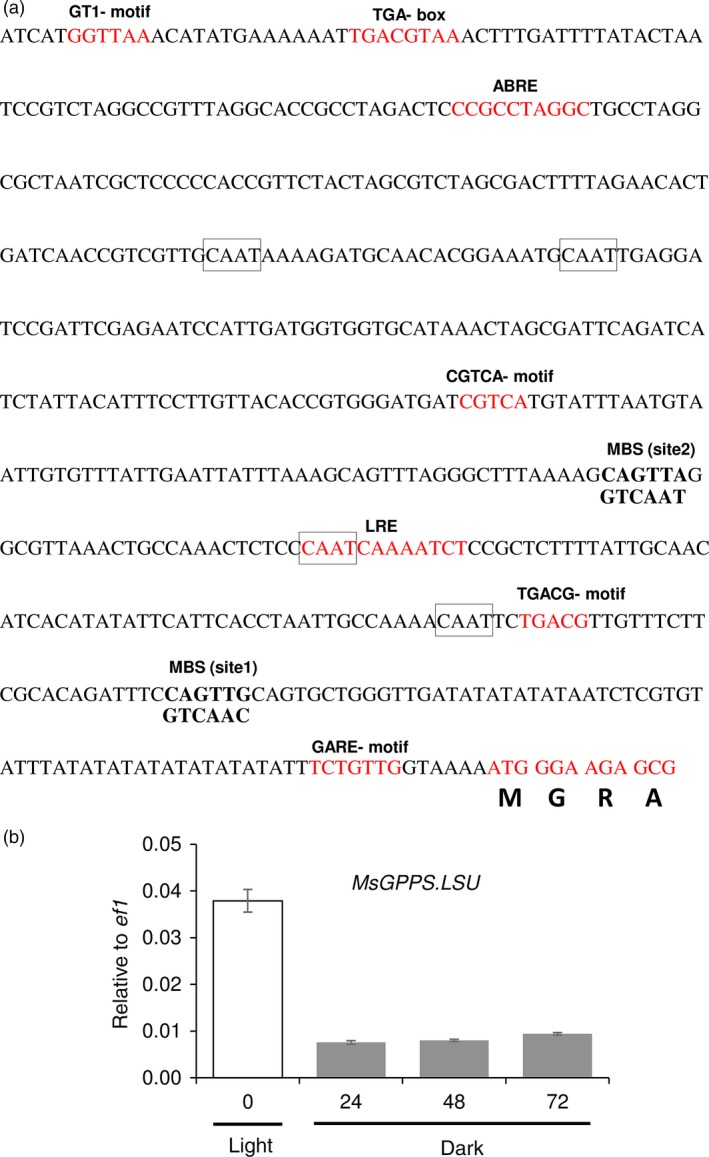
Promoter analysis of *MsGPPS*.*LSU*. (a) Regulatory elements present in the promoter region (−549 bp) of *MsGPPS*.*LSU*. (b) Decreased levels of *MsGPPS*.*LSU* in plants grown under dark when compared to plants grown under light. Data are indicated as mean ± SE.

To understand how MsMYB affects terpene metabolism, it is essential to know its downstream target genes. Towards this, we checked the expression levels of several transcripts encoding enzymes in various terpene precursor pathways, transporters and flavonoid pathway (Table S2), but none of them were significantly changed except for *MsGPPS.LSU*. It showed increased levels in *MsMYB*‐RNAi lines and decreased levels in *MsMYB*‐overexpressing lines (Figure 6a,b). GPPSs are known to localize to both nongreen plastids and chloroplasts of photosynthetic cells where MEP pathway is active (Bouvier *et al*., 2000). We checked the localization of MsGPPS.LSU and found it to be localized in the chloroplast (Figure 9b). To check whether the promoter of *MsGPPS.LSU* has putative MYB binding sites, we cloned a 549‐bp genomic DNA fragment upstream of the translation start site by genome walking. Using PlantCARE tool we identified two MYB binding sites (MBS) along with several elements for hormones and light responsiveness (Figure 7a).

**Figure 8:**
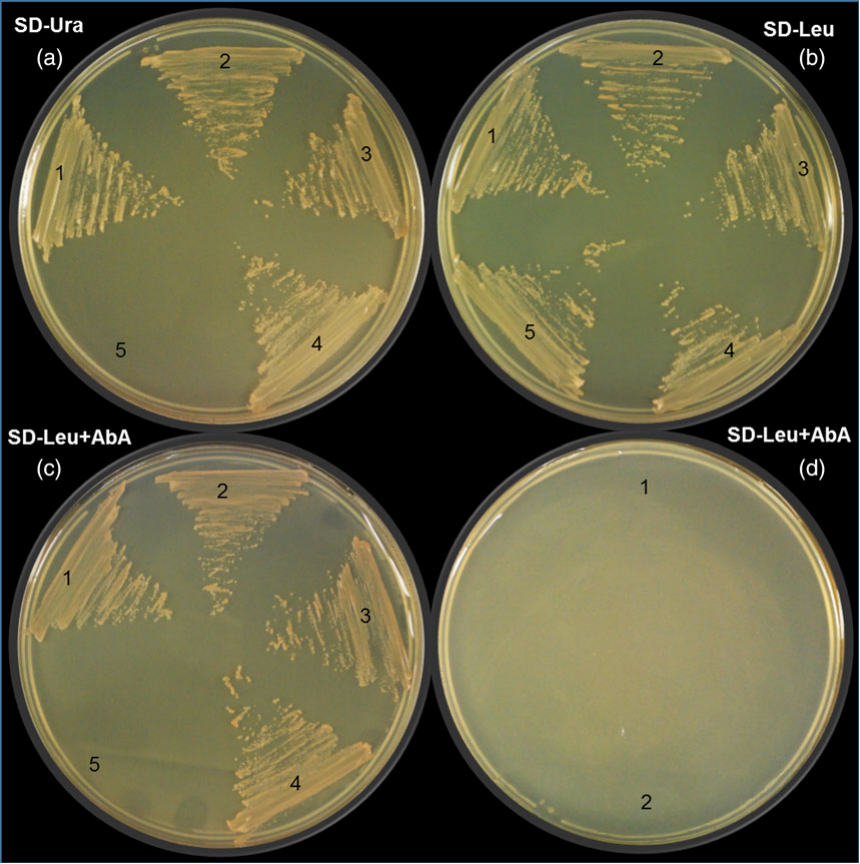
Yeast one‐hybrid assay between MsMYB and *GPPS*.*LSU* promoter. (a) Bait strains of (1) pAbAi‐GPPS‐full, (2) pAbAi‐site1, (3) pAbAi‐site2, (4) pAbAi‐p53 (positive control) and (5) auxotrophic Y1HGold (negative control) growing on SD/‐Ura. (b) pGADT7‐MYB prey expression in (1) pAbAi‐GPPS‐full, (2) pAbAi‐site1 and (3) pAbAi‐site2; pGADT7‐p53 prey expression in (4) pAbAi‐p53 and empty pGADT7 in (5) pAbAi‐p53 on SD/‐Leu. (c) DNA–protein interaction of (1) full *GPPS* promoter and MYB, (2) site 1 and MYB, (3) site 2 and MYB, (4) pAbAi‐p53 and pGADT7‐p53, and (5) no interaction of pAbAi‐p53 and empty pGADT7 on SD/‐Leu/AbA. (d) Absence of DNA–protein interaction of (1) pAbAi‐mut‐site1 and (2) pAbAi‐mut‐site2 with MYB. SD/‐Ura, SD medium without Ura; SD/‐Leu, SD medium without Leu; SD/‐Leu/AbA, SD medium without Leu but containing aureobasidin A (800 ng).

**Figure 9:**
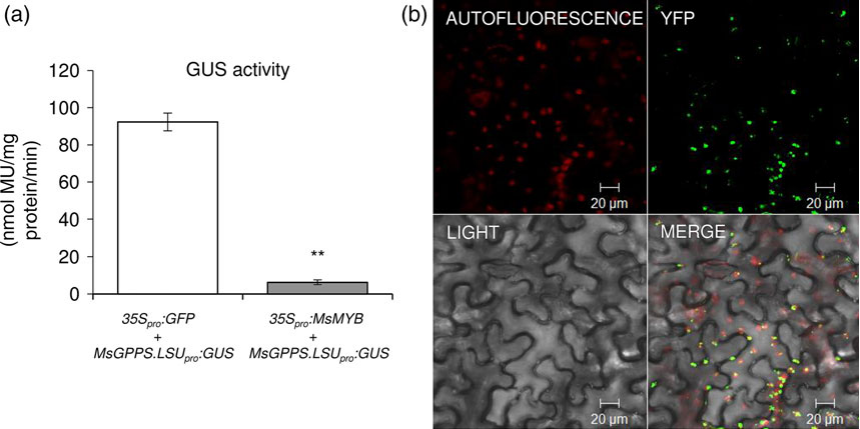
Transcriptional repression activity of MsMYB and localization of MsGPPS.LSU. (a) Reduced levels of GUS in *35S*_*pro*_ *:MsMYB* expressing leaves (b) Chloroplast‐specific localization of MsGPPS.LSU. Data are indicated as mean ± SE.

To verify MsMYB interaction with *GPPS.LSU* promoter, yeast one‐hybrid assays were performed. The whole *GPPS.LSU* promoter along with two truncated regions, viz. site 1 and site 2, which had the conserved MYB binding domains and mutated versions of site 1 and site 2 was used as bait to generate five Y1HGold [Bait/AbAi] strains, that is pAbAi‐GPPS‐full, pAbAi‐site1, pAbAi‐site2, pAbAi‐mut‐site1 and pAbAi‐mut‐site2. After cotransformation with functional *MsMYB* gene in pGADT7 vector (pGADT7‐MYB), three Y1HGold strains, that is pAbAi‐GPPS‐full, pAbAi‐site1 and pAbAi‐site2, could grow on SD minus leucine with aureobasidin (SD/‐Leu/AbA, 800 ng of AbA) auxotrophic medium but not the two mutant strains pAbAi‐mut‐site1 and pAbAi‐mut‐site2, confirming interaction of MsMYB with *GPPS.LSU* promoter *in vivo* (Figure 8). TFs can act as activators or repressors of transcription. Transgenic studies indicated that *MsMYB* is a negative regulator. To further determine the transcriptional repression activity of MsMYB in planta, transient expression assay in *N. benthamiana* was performed. We used *35S*_*pro*_ *:GFP* or *35S*_*pro*_ *:MsMYB* as effectors and *MsGPPS.LSU*_*pro*_ *:GUS* as reporter. Promoter activity of *MsGPPS.LSU*_*pro*_ *:GUS* was significantly reduced in *35S*_*pro*_ *:MsMYB* expressing leaves when compared to *35S*_*pro*_ *:GFP* expressing leaves (Figure 9a). These results show that *MsMYB* binds to the promoter of *MsGPPS.LSU* and suppresses its activity.

Terpene biosynthesis is known to be regulated by light in many plants (Cordoba *et al*., 2009). As we found light‐responsive elements in *MsGPPS.LSU* promoter, we checked whether *MsGPPS.LSU* is also regulated by light. WT spearmint plants were grown in dark and light conditions. Leaf samples were collected at 0‐, 24‐, 48‐ and 72‐h interval to quantify *MsGPPS.LSU* transcript levels. Expression of *MsGPPS.LSU* was highly reduced in the plants grown under dark conditions suggesting that *MsGPPS.LSU* is regulated by light (Figure 7b). As GPPS is a key enzyme in the monoterpene pathway, multiple layers of gene regulation to fine tune the pathway is plausible.

### Ectopic expression of *MsMYB* affects secondary terpene metabolism in sweet basil and *Nicotiana sylvestris*

**Figure 10:**
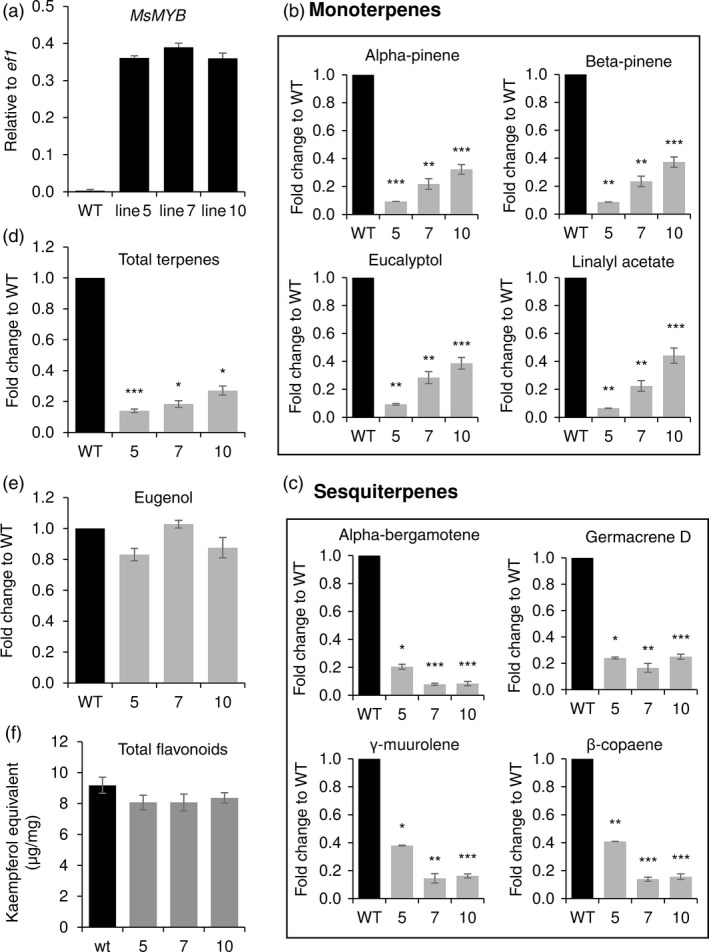
Ectopic expression of *MsMYB* in sweet basil alters the amount of secondary metabolites. (a) *MsMYB* expression in basil transgenic plants. (b), (c) Reduced levels of monoterpenes and sesquiterpenes. (d) Decreased levels of total terpenes. (e) Unaltered levels of phenylpropenes (eugenol). (f) Unaltered levels of total flavonoids. Data are indicated as mean ± SE. *P < 0.05; **P < 0.01; ***P < 0.001.

Similar to spearmint, sweet basil too produces its essential oil in PGTs. The constituents of sweet basil essential oil are derived from terpene and phenylpropanoid pathways (Figure S3b). It is a mixture of monoterpenes, sesquiterpenes and the main phenylpropanoid derivative is eugenol. Ectopic expression of *MsMYB* in sweet basil was pursued to evaluate whether *MsMYB* can affect sesquiterpene pathway and phenylpropanoid pathway as suggested by phylogenetic analysis. Three independent lines (line 5, line 7 and line 10) confirmed by Southern blot were further selected for characterization (Figure S1d). Ectopic expression of *MsMYB* in these lines was confirmed by qRT‐PCR (Figure 10a). GC‐MS analysis on T‐2 plants revealed an overall 73%–85% decrease in total terpenes, both monoterpenes and sesquiterpenes (Figure 10b,c,d). However, the amount of eugenol derived from phenylpropanoid pathway remained unaltered (Figure 10e). Additionally, the leaves of transgenic lines were smaller in size when compared to WT (Figure S4a,b). From the NCBI database, we obtained the sequences of sweet basil geranyl diphosphate synthase large subunit (*ObGPPS.LSU*) and farnesyl diphosphate synthase (*ObFPPS*). To investigate whether similar to spearmint, the decrease in terpenes in sweet basil is due to the reduction in transcripts of *ObGPPS.LSU* or because of reduction in *ObFPPS* transcript, we measured their levels by qRT‐PCR. When compared to WT, *ObGPPS.LSU* expression was significantly reduced in transgenic plants (Figure 6c), but levels of *ObFPPS* were unaltered. Additionally, we measured the total flavonoid content in the leaves of sweet basil transgenic plants and found the total flavonoid content to be unaltered (Figure 10f). This suggests that MsMYB is a R2R3‐MYB specific to terpene metabolism.

The sweet basil data showed that *MsMYB* can affect both monoterpene and sesquiterpene synthesis. To investigate whether it has a role in diterpene synthesis, we ectopically expressed *MsMYB* in *N. sylvestris*. Glandular trichomes of *N. sylvestris* mainly produce diterpenes; cembranoids (CBT‐diol) are generally derived from the same MEP pathway as the monoterpenes. Three independent lines were chosen for further characterization and ectopic expression of *MsMYB* was confirmed by qRT‐PCR (Figure S5a). Analysis of CBT‐diols production in transgenic plants showed a 42%–50% reduction (Figure S5b). Similar to sweet basil plants, the leaves of tobacco transgenic lines were smaller in size when compared to WT (Figure S4c). The above results indicate that *MsMYB* can affect the flux in various branches of terpene metabolism.

### *MsMYB* is probably not a target of microRNA858

**Figure 11:**
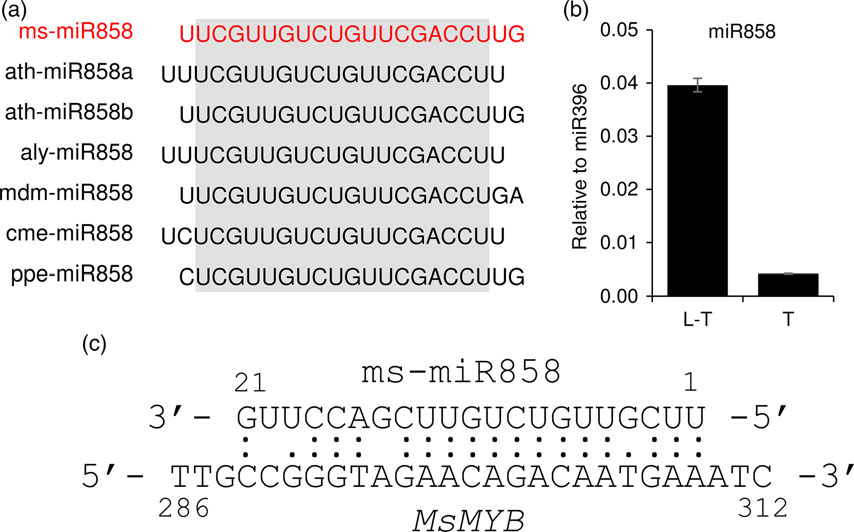
ms‐miR858 analysis. (a) ms‐miR858 sequence comparison with other known miR858 sequences from various species. miR858 sequences were obtained from miRBase. ms—*Mentha spicata*; ath—*Arabidopsis thaliana*; aly—*Arabidopsis lyrata*; mdm—*Malus domestica*; cme—*Cucumis melo*; ppe—*Prunus persica*. (b) Expression levels of ms‐miR858 in leaf stripped of PGTs (L‐T) and PGTs (T). (c) Probable binding of ms‐miR858 to *MsMYB*.

microRNA858 is known to regulate several R2R3‐MYBs, specifically the subgroup 7 R2R3‐MYBs, *AtMYB11, AtMYB111* and *AtMYB12* (Fahlgren *et al*., 2007; Sharma *et al*., 2016). As MsMYB fell under subgroup 7, we were interested to know whether it is regulated by microRNA858. We generated small RNA sequencing data from PGTs and L‐PGT and identified ms‐miR858. The sequence similarity of ms‐miR858 is shown in Figure 11a. Expression analysis from the sequencing data showed that ms‐miR858 had higher expression in L‐PGT tissue when compared with PGT. This was validated by TaqMan^®^ qPCR assay using miR396 as internal control due to its stable expression in L‐PGT and PGT tissues (Figure 11b). The Plant Small RNA Target Analysis Server (Dai and Zhao, 2011) predicted *MsMYB* as a probable target of ms‐miR858 (Figure 11c). To test whether low levels of miR858 expression can target *MsMYB*, we looked for cleavage products through RNA ligase‐mediated 5′ amplification of cDNA ends (RLM‐RACE) and poly(A) polymerase‐mediated (PPM)‐RACE. However, no cleavage products of *MsMYB* were found in our conditions, thus reducing the possibility of it being regulated by miR858. *MsMYB* and miR858 appears to be differentially regulated at transcriptional level making their interaction less feasible.

## Discussion

Extensive exploitation of aromatic plants for their commercially valued secondary metabolites calls for novel strategies for metabolic engineering to increase yield. A complete understanding of the secondary metabolism pathway by unravelling the enzymes and TFs controlling the pathway is indispensable for the same. PGTs are found on the aerial parts of many aromatic plants but very few studies have focused on identifying regulators of secondary metabolite production from these organs (Wang, 2014). In this study, we identified and characterized a R2R3‐MYB gene, *MsMYB* from spearmint which is highly expressed in PGTs and acts as a negative regulator of monoterpene synthesis. GPPS enzyme is responsible for the formation of monoterpene precursor C_10_‐GPP in plastids and regulates flux into this pathway. Details about their function and regulation in plants are very scarce. The heteromeric GPPS in spearmint consists of a small and large subunit and interaction between them results in an active GPPS. Structural and mutagenic studies of these two subunits have shown that the large subunit is catalytically active and presumably through protein–protein interactions, and the SSU shapes the active site cavity of LSU towards producing C_10_‐GPP and restricts the production of long‐chain C_15_ or C_20_ prenylphosphates (Chang *et al*., 2010). In spearmint, both the subunits of GPPS are preferentially expressed in PGTs (Turner and Croteau, 2004). MsMYB was found to interact with *MsGPPS.LSU* promoter and repress its activity. The increased production of monoterpene in *MsMYB*‐RNAi lines can be attributed to enhanced production of GPP through greater production of MsGPPS.LSU. Misexpression of either the regulatory or catalytic subunit are known to alter enzyme activity in plants and animals (Gergs *et al*., 2004; He *et al*., 2004; Hu *et al*., 2016; Tiessen *et al*., 2002). In snapdragon and hop where heteromeric GPPS are found, only GPPS.SSU is closely correlated with monoterpene production, whereas the GPPS.LSU is more ubiquitous. But spearmint *GPPS.LSU* expression is more PGT‐specific (Jin *et al*., 2014). Unlike the purified mint GPPS.LSU, which is inactive by itself, the potential GPPS.LSU from snapdragon and hops are active alone and functions as GGPPS (Tholl *et al*., 2004; Wang and Dixon, 2009). This suggests function of GPPS subunits from different plants might vary and also the mechanisms that regulate them.

Ectopic expression of *MsMYB* in sweet basil and tobacco revealed that it could also perturb metabolites derived from sesquiterpene and diterpene pathways. Sesquiterpenes and diterpenes are derived from C_15_‐FPP and C_20_‐GGPP which are produced in cytoplasm and plastids through MVA and MEP pathway, respectively. Studies have demonstrated that exchange of precursor metabolites can occur between the cytosolic MVA and plastidial MEP pathway. Metabolic intermediates like IPP, GPP and GGPP can be transported across plastidial membranes through transporters (Hemmerlin *et al*., 2012; Vranova *et al*., 2013). FPPS catalyses the sequential condensation of two molecules of IPP with DMAPP to form C_15_‐FPP, the immediate precursor for all sesquiterpenes (Cornish, 1993; Lange *et al*., 2000). But FPPS is also know to accept C_10_‐GPP as an allylic substrate to generate FPP (Guo *et al*., 2015; Hemmerlin *et al*., 2003). Further sesquiterpenes synthesized in cytoplasm can be derived from plastidial GPP too (Adam *et al*., 1999). Hence, an alteration in GPP produced in plastids can have an effect on sesquiterpene production. Ectopic expression of *MsMYB* in sweet basil resulted in reduced expression of *ObGPPS.LSU*. The decrease in sesquiterpene production observed in sweet basil indicates that GPP produced might be used towards synthesizing sesquiterpene especially as no change was observed on sweet basil FPPS. The monoterpene and diterpene pathway are both largely localized to plastids. GGPPS is responsible for producing GGPP for diterpenes (C_20_) and tetraterpenes (C_40_) synthesis. Apart from IPP and DMAPP, *in vitro* data show that GPP or FPP can also be used by GGPPS as allylic substrates to generate GGPP (Hefner *et al*., 1998; Okada *et al*., 2000; Takaya *et al*., 2003). The decrease in diterpenes produced in *MsMYB*‐overexpressing tobacco plants can be due to a reduction in endogenous pool of GPP. Tobacco generally produces low levels of monoterpenes and there is no information currently about endogenous GPPS from it. Although introduction of monoterpene synthases from other plants does result in the emission of volatile monoterpenes in tobacco, suggesting some amount of GPP availability (Lücker *et al*., 2004). Apart from alteration in terpene‐derived secondary metabolites, overexpression of *MsMYB* in tobacco and sweet basil generated smaller leaves. Several major plant hormones are produced through terpene pathway such as gibberellin, cytokinin, abscisic acid and strigolactone which control various aspects of plant development (Gomez‐Roldan *et al*., 2008; Umehara *et al*., 2008). Further experiments need to be carried out to identify perturbation in which hormone/hormones leads to smaller leaf size. This phenotype was not observed in spearmint. TFs can have additional targets when ectopically expressed. For example, maize ZmMYB31 cannot bind to the promoter of maize *4CL* and *C3H* genes; however, it can repress the expression of Arabidopsis *4CL* and *C3H* when overexpressed in Arabidopsis (Fornalé *et al*., 2010). There is a possibility that additional target genes related to primary terpene metabolism are regulated when MsMYB is overexpressed heterologously in these plants. Ectopic expression of *MsMYB* did not lead to any observable defect in chlorophyll formation which is a primary metabolite derived from diterpenes. An earlier study showed that GGPP used in chlorophyll biosynthesis is formed directly from DMAPP and IPP rather than from GPP and IPP. Thus, any alterations in the amount of GPP formed do not affect chlorophyll biosynthesis (Van Schie *et al*., 2007).

TFs are known to regulate multiple genes. *MsGPPS.LSU* is probably one of the many downstream targets regulated by MsMYB. Hence, we cannot negate the fact that the effects seen on terpene metabolism in spearmint, sweet basil and tobacco can be due to other genes as well. Transcriptome analysis of *MsMYB*‐RNAi lines can provide us with more candidate genes that are potentially regulated by *MsMYB*. We found that *MsGPPS.LSU* is further regulated by light. Several genes of MEP pathway are known to be regulated by light (Cordoba *et al*., 2009). Secondary metabolite production is mainly for plants biotic and abiotic stress responses, so stringent multiple regulatory mechanisms involving activators and repressors must exist to determine the timing, amount and patterning of these compounds.

Majority of R2R3‐MYBs characterized in plants are involved in the regulation of different classes of phenylpropanoid‐derived compounds (Liu *et al*., 2015). Phylogenetic analysis showed that MsMYB is very similar to subgroup 7 of Arabidopsis R2R3‐MYBs which include MYB11, MYB12 and MYB111. These MYBs have been characterized as activators of biosynthetic enzymes of flavonoid pathway which is derived from the general phenylpropanoid pathway. Analysis of major flavonoid pathway genes from spearmint showed no change in *MsMYB* transgenic lines. Further no change was seen in either eugenol production which is derived from phenylpropanoid pathway or in total flavonoid content of leaves from sweet basil *MsMYB‐*overexpressing lines. These results suggest *MsMYB* as a regulator of terpene metabolism and not affecting flavonoid production. This indicates towards diversification of function within the same subgroup. Although the N‐terminus of R2R3‐MYBs is conserved, C‐terminus amino acids are very diverse and unique providing opportunity for divergence. Comparison of MYB proteins from different species will furnish new insights into the evolution of this important family of TF. In the past few years numerous studies have demonstrated miR858 can target R2R3‐MYBs from various plants including the members of subgroup 7 of Arabidopsis R2R3‐MYBs (Addo‐Quaye *et al*., 2008; Rock, 2013; Sharma *et al*., 2016; Xia *et al*., 2012). Small RNA‐Seq data of spearmint PGTs and L‐PGTs showed an inverse pattern of expression for miR858 and *MsMYB*. High expression was seen in L‐PGTs and low in PGTs for miR858 which is opposite to *MsMYB* expression, indicating a differential transcriptional regulation. *MsMYB* promoter is able to drive trichome specific expression as seen in tobacco. As genome of spearmint is not available, we cannot verify the promoter activity of miR858. Although *in silico* analysis predicts *MsMYB* to be a potential target for miR858, no cleavage products could be observed. This suggests that the PGT specific expression of *MsMYB* is governed mainly by the *cis*‐elements present in its promoter and may be by other *trans*‐factors but not by miR858.

In conclusion, *MsMYB* is the first R2R3‐MYB gene identified from spearmint that is related to terpene secondary metabolism. MsMYB represses *MsGPPS.LSU* and is able to modify terpene production. This opens up new avenues and targets to metabolically engineer plants for altering pathways.

## Experimental procedures

### Plant material and transformation

Secondary metabolites of commercial spearmint variety (*M. spicata*) and sweet basil (*O. basilicum*) were analysed by GC‐MS and grown in green house under natural light conditions. *Agrobacterium* mediated transformation of spearmint was performed according to previously published protocol (Niu *et al*., 1998, 2000) with several modifications. The complete details of transformation are provided in the supplementary data. *Agrobacterium* mediated transformation of sweet basil was performed as previously described by Wang *et al*. (2016). Tobacco transformation was performed as described previously (Gallois and Marinho, 1995).

### RNA isolation and quantitative real‐time PCR (qRT‐PCR)

For PGT RNA isolation, initially, PGTs were isolated from 2‐ to 3‐cm leaves as described previously (Jin *et al*., 2014). Later, total RNA was extracted from PGT using the Spectrum Plant total RNA kit from Sigma (UK). Total RNA from other tissues was extracted using an RNeasy^®^ Plus Mini kit from Qiagen (Germany). 500 ng of RNA was reverse‐transcribed to cDNA using iScript™ cDNA Synthesis kit form Bio‐Rad (CA, USA). Expression levels of genes along various tissues were analysed using qRT‐PCR that was carried out as described previously (Jin *et al*., 2014). In current study, elongation factor 1 (*ef1*) was used as internal control for both spearmint and sweet basil, due to its stable expression in plant (Nicot *et al*., 2005). For tobacco, elongation factor TuB (EF‐TuB) was used as internal control. Error bars represent mean ± SE.

### TaqMan^®^ qPCR assay

7 ng of total RNA was reverse‐transcribed using the TaqMan^®^ microRNA reverse transcription kit (Applied Biosystems, USA) according to the manufacturer's protocol. Custom‐made primers were used specifically for miR858 and miR396 cDNA synthesis. Custom TaqMan^®^ small RNA assays (Applied Biosystems, USA) were carried out for microRNA qPCR validation using custom‐made TaqMan^®^ probe for miR858 and miR396. Expression values were calculated relative to the internal control miR396.

### Promoter cloning and analysis

Genomic DNA was isolated from young leaves of spearmint using CTAB method. The flanking sequences of *MsMYB* and *MsGPPS.LSU* genes were amplified using a GenomeWalker™ Universal kit (Clontech, USA) and later ligated to pGEM^®^‐T vector. The resulting product was transformed into *Escherichia coli* (*E. coli*) XL1‐Blue cells and sequenced. The promoter was amplified with Phusion^®^ High‐Fidelity DNA Polymerase (NEB, USA) and subcloned into a gateway donor vector pENTR^™^/D‐TOPO^®^ (Invitrogen, Germany). Further, the recombinant plasmid was introduced into destination vector pBGWFS7 by LR recombination. The destination plasmid was further transformed into *Agrobacterium* EHA105 by heat shock. The recombinant *Agrobacterium* EHA105 strain was used to generate transgenic tobacco lines.

### Gene amplification and plasmid construction

Full‐length ORF encoding MsMYB was obtained by performing 3′ and 5′ RACE using the SMARTer™ RACE cDNA amplification kit from Clontech (CA, USA). For sequencing of ORF, purified fragments were ligated with pGEM^®^‐T vector and transformed into *E. coli* XL1‐Blue cells. To overexpress *MsMYB*, firstly, the sequences were amplified with Phusion^®^ High‐Fidelity DNA Polymerase (NEB, USA). For *MsMYB‐*RNAi, four primers with restriction enzymes located at flanking region were used to amplify the fragment showing low similarity to other *MYB* genes. The purified PCR products were then cloned into the donor vector and subsequently introduced into the destination vector pK7WG2D via LR recombination. The *MsMYB* gene was driven by 35S promoter in both overexpression and RNAi plants. All destination plasmids harbouring target gene were transformed into *Agrobacterium* EHA105 by heat shock. The recombinant *Agrobacterium* EHA105 strains were used for plant transformation. Sequences of primers used in this study are listed in Table S1.

### Subcellular localization of TFs

The full‐length cDNA of *MsMYB* without the stop codon was cloned into the gateway vector pENTR/D‐TOPO (Invitrogen, Germany) and then subsequently transferred into the destination vector pBA‐DC‐YFP (Zhang *et al*., 2005) which contains the CaMV 35S promoter and C‐terminal in frame with YFP, to generate *MsMYB‐YFP*. The construct was then introduced into *Agrobacterium* strain EHA105 by heat shock. 4′,6‐Diamidino‐2‐phenylindole (DAPI) was used as maker to stain nucleus. *N*. *benthamiana* seeds were germinated on MS plate and transferred into soil. Three weeks after growing in the glasshouse, the seedlings were used for agroinfiltration. Subcellular localization was performed as described by Wang *et al*. (2016).

### Selection of transgenic lines

Initially, visual screening using GFP filter was pursued to isolate GFP‐positive plants. Later, DNA was isolated from GFP‐positive plants and insertion of gene of interest was checked using PCR. DNA‐positive lines were then subjected to Southern blot using DIG wash and block buffer set from Roche (IN, USA). DNA probe against CaMV 35S promoter was generated using PCR DIG probe synthesis kit from Roche (IN, USA) (Hart and Basu, 2009). Concentration of probe was quantified by creating a dot plot using DIG nucleic acid detection kit from Roche (IN, USA) as described previously (Javelle *et al*., 2011). A total of 15 μg genomic DNA was digested overnight with *NdeI* at 37 °C. The next day, digested product was electrophoresed on a 1% (w/v) agarose gel at 40 V for 5 h. After that, the gel was transferred to a nylon membrane and hybridized with CaMV 35S promoter probe followed by antibody binding and chemiluminescent reaction development by CDP‐Star. Finally, the membrane was exposed in a film and the number of T‐DNA insertions were analysed. Spearmint plants were clonally propagated thrice before analysis. Basil plants were analysed in T2 generation and a minimum of six plants were sampled for each line. Tobacco plants were analysed in T1 generation. For each line, ~32 plants were used for phenotypical screening and six plants for GC‐MS analysis.

### GC‐MS and total flavonoid content analysis

For GC‐MS studies, spearmint plants were clonally propagated three times before collecting about 4–6 leaves of 2–3 cm which were ground to a fine powder using liquid nitrogen and homogenized using 500 μL ethyl acetate. Camphor was added as an internal control. For sweet basil, the analysis was performed in T‐2 plants. From bottom, leaves of 3–4 cm at fourth node were used. For tobacco, 4‐ to 5‐cm leaves were used from T1 plants. Diethyl sebacate was added as an internal standard in sweet basil and tobacco samples. Samples were incubated for 10 min at room temperature with vigorous shaking followed by a centrifugation for 10 min at 15871***g***. The top organic layer was transferred to a new tube and dehydrated using anhydrous Na_2_SO_4._ The samples were analysed using GC‐MS (7890A with 5975C inert MSD with triple axis detector, Agilent Technologies, USA). 2 μL of samples was injected, and separation was achieved with a temperature programme of 50 °C for 1 min and increased at a rate of 8 °C/min to 300 °C and held for 5 min, on a 30 m HP‐5 MS column (Agilent Technologies, USA). Estimation of total flavonoid content was performed using the aluminium chloride (AlCl_3_) colorimetric method as described previously (Vaidya *et al*., 2014).

### Yeast one‐hybrid assay

For ascertaining the interaction between MsMYB and *MsGPPS.LSU* promoter, a Matchmaker Gold yeast one‐hybrid library screening system (Clontech, USA) was used. As *MsGPPS.LSU* promoter encompasses two different MYB binding sites, two promoter regions approximately 40–45 bp in size harbouring the two sites, namely site 1 and site 2, were cloned into the MCS of pAbAi vector independently in addition to two mutant versions of these two sites. Furthermore, complete promoter was also cloned in pAbAi vector and used as bait for further confirmation. The *MsMYB* gene was cloned into the pGADT7 vector to be used as prey in the form of functional fusion protein along with GAL4 transcription activation domain (GAL4 AD). As a positive control, p53 bait and prey provided in the kit was used. Mutated site 1 and mutated site 2 were used as negative control. Yeast transformation and validation of positive interactions were implemented as described in the Matchmaker Gold Yeast one‐hybrid system user manual (www.clontech.com/xxclt_ibcGetAttachment.jsp?cItemId=17599). The primers used are listed in Table S3.

### Transactivation activity assay

The 549‐bp promoter region of *MsGPPS.LSU* was amplified and inserted into pENTR^™^/D‐TOPO^®^. The resulting plasmid was transformed into pBGWFS7 by LR recombination and further introduced into *Agrobacterium* EHA105. Leaves of *N. benthamiana* were agroinfiltrated with effector and reporter at a ratio of 1:1. Two days after infiltration, leaves were harvested to isolate crude protein. GUS quantitative assay was performed in triplicate as described in Li *et al*. (2014).

### RLM‐RACE and PPM‐RACE

For RLM‐RACE, 5′ RACE adapter was ligated to 1 μg of total RNA, and for PPM‐RACE, poly(A) tailing of 1 μg of total RNA was performed using poly(A) polymerase (Invitrogen, Germany). RNA was then reverse‐transcribed and amplified for 5′ and 3′ ends using FirstChioce RLM‐RACE kit (Thermo Fisher, USA) according to the manufacturer's protocol.

### Small RNA library construction and high‐throughput sequencing

The RNA libraries were prepared using the TruSeq RNA library prep Kit v2, set A (Illumina Inc., USA) according to manufacturer's instructions. The quality and size of DNA libraries for sequencing were checked using the Agilent 2200 TapeStation system. Three libraries were run on single lanes on HiSeq^™^ 2000 (Illumina Inc., USA), individually.

### Bioinformatics analysis of sequencing data

After removing adapters by Cutadapt tool (Martin, 2011), modified sequences from 18 nt to 30 nt were used for further analysis. To begin with, sequences of rRNAs, tRNAs, snRNAs and snoRNAs available in Rfam11.0 database (ftp://ftp.ebi.ac.uk/pub/databases/Rfam/11.0/) were removed from the small RNA sequence reads. Deposited miR858 sequences from miRBase database (http://www.mirbase.org/search.shtml) were mapped to the remaining small RNA sequence reads by bowtie tool (Langmead and Salzberg, 2012) to identify ms‐miRNA858. A maximum of one mismatch per sequence was set as a parameter.

### Phylogenetic analysis

Phylogenetic tree was constructed using MEGA6 software by the neighbour‐joining method with bootstrap values of 1000 replicates and edited in FigTree. *A*. *thaliana* R2R3‐MYB sequences were obtained from TAIR website.

### Statistical analysis

Data are indicated as ‘mean ± SE’ of three to six biological replicates each performed in triplicate. Statistical significance between transgenic plants and WT was analysed using a two‐tailed Student's *t*‐test and indicated by asterisks. * indicates *P* < 0.05; ** indicates *P* < 0.01; *** indicates *P* < 0.001.

### Accession numbers

Sequence data of *A. thaliana* R2R3‐MYBs used in Figure S2 can be found in TAIR library under accession numbers listed in Supplemental Data S2. Sequence data of *ObGPPS.LSU* and *ObFPPS* can be found in NCBI database under the sequence ID DY340136 and DY332783, respectively. Sequence data of *MsMYB* have been deposited in GenBank under the accession number KY081780.

## Competing interests

The authors declare that they have no competing interests.

## Supporting information


**Figure S1.** Scanning electron micrograph (SEM) of leaf surface and Southern blot analysis of transgenic plants.
**Figure S2.** Phylogenetic tree showing the similarity of MsMYB to known *Arabidopsis thaliana* R2R3‐MYBs.
**Figure S3.** GC profiles of wild‐type plants.
**Figure S4.** Transgenic plants overexpressing *MsMYB* show smaller leaf size.
**Figure S5.** Ectopic expression of *MsMYB* in tobacco.
**Table S1.** Primers used in this study.
**Table S2.** List of genes analysed in spearmint transgenic plants.
**Table S3.** Oligonucleotides used for the generation of bait sequences.

**Data S1.** Sequence data and expression values of spearmint R2R3‐MYBs.

**Data S2.** Accession numbers of *A. thaliana* R2R3‐MYBs.
