# Supplementary material for: Spearmint R2R3‐MYB transcription factor MsMYB negatively regulates monoterpene production and suppresses the expression of geranyl diphosphate synthase large subunit (MsGPPS . LSU )
Source: Plant Biotechnol J. 2017 Mar 18;15(9):1105–19. doi: 10.1111/pbi.12701 (PMC5552485; doi:10.1111/pbi.12701)
Supplement: Supplementary file 1 — Figure S1. Scanning electron micrograph (SEM) of leaf surface and Southern blot analysis of transgenic plants. Figure S2. Phylogenetic tree showing the similarity of MsMYB to known Arabidopsis thaliana R2R3‐MYBs. Figure S3. GC profiles of wild‐type plants. Figure S4. Transgenic plants overexpressing MsMYB show smaller leaf size. Figure S5. Ectopic expression of MsMYB in tobacco. Table S1. Primers used in this study. Table S2. List of genes analysed in spearmint transgenic plants. Table S3. Oligonucleotides used for the generation of bait sequences. [file PBI-15-1105-s003.pdf]

(a)

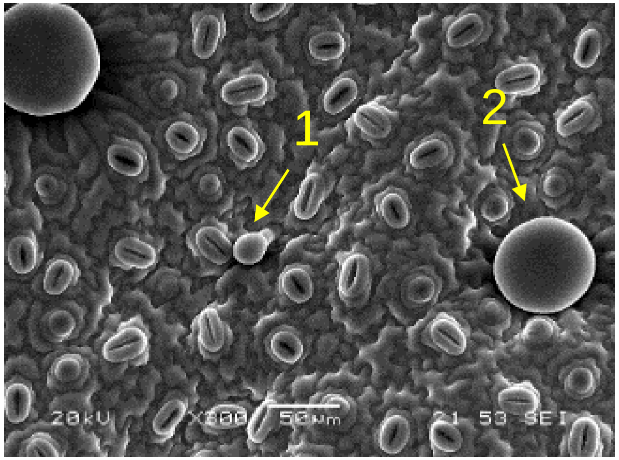

(b)

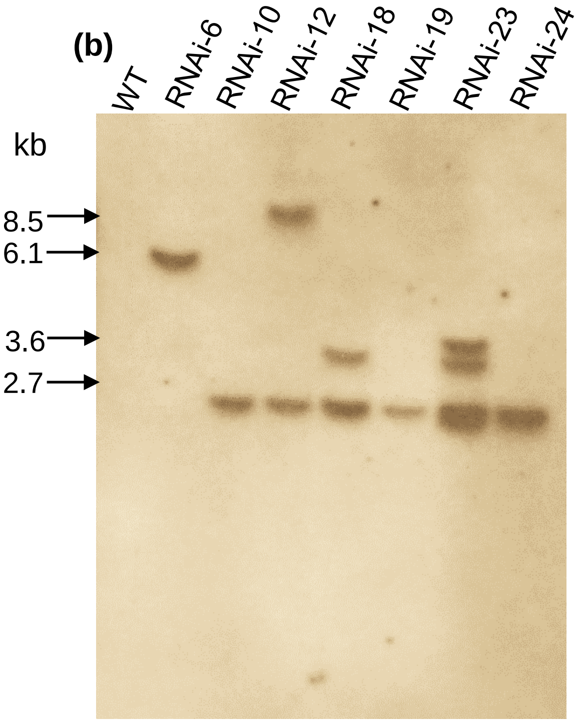

(c)

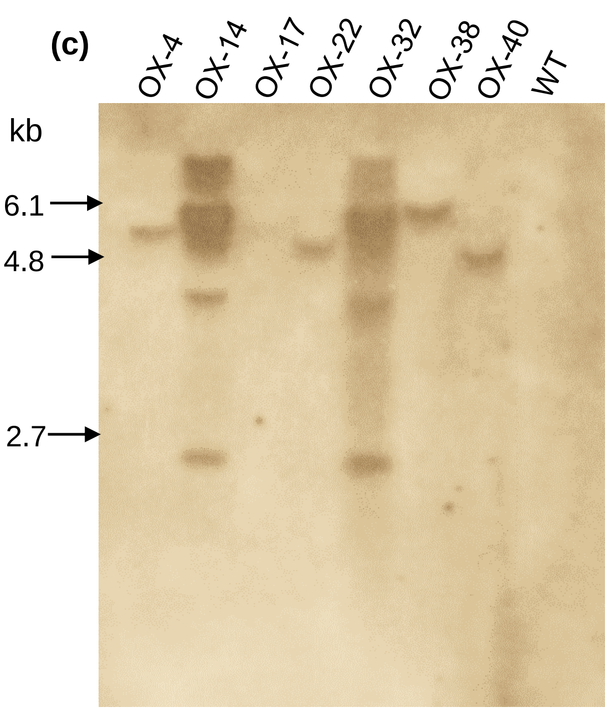

(d)

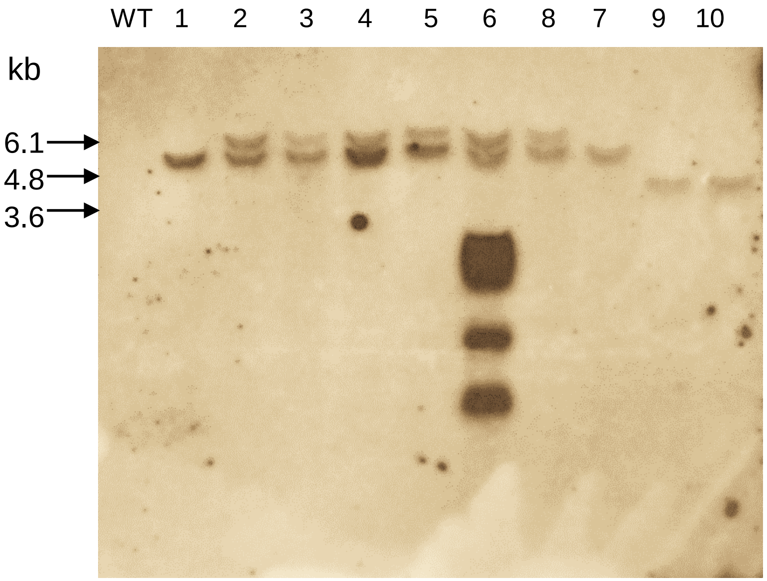

Figure S1. Scanning electron micrograph (SEM) of leaf surface and southern blot analysis of transgenic plants. (a) SEM of a spearmint leaf showing two kinds of glandular trichomes, (1) capitate glandular trichome and (2) peltate glandular trichome. (b), (c) Southern blot of *MsMYB*-RNAi and *MsMYB*-overexpressing spearmint lines showing a range of insertions. (d) Southern blot of transgenic sweet basil lines overexpressing *MsMYB* showing different T-DNA insertions. 15µg of DNA was digested with NdeI enzyme.

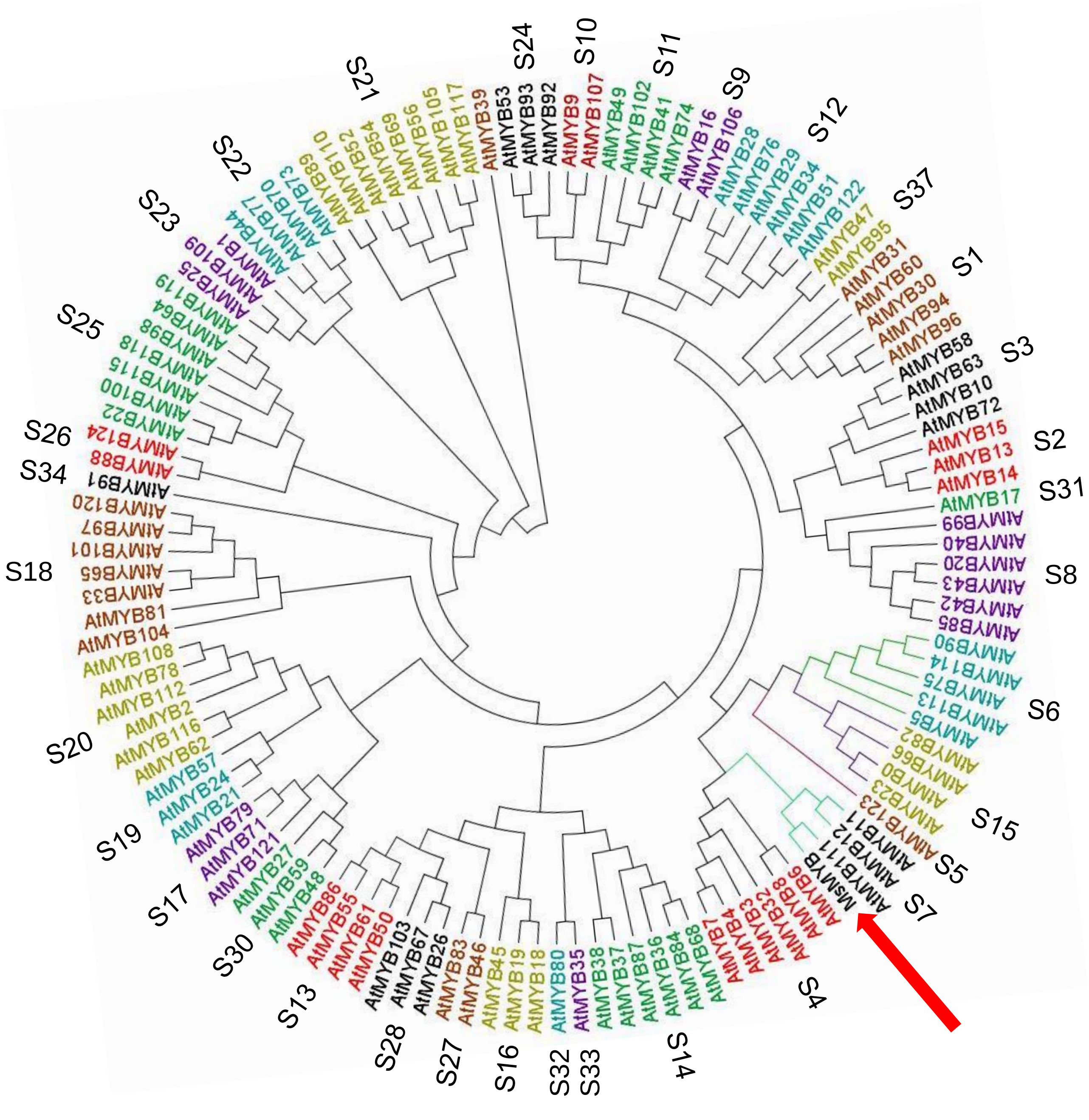

Figure S2. Phylogenetic tree showing the similarity of MsMYB to known *Arabidopsis thaliana* R2R3-MYBs. MsMYB is pointed with a red arrow. MsMYB falls under subgroup 7.

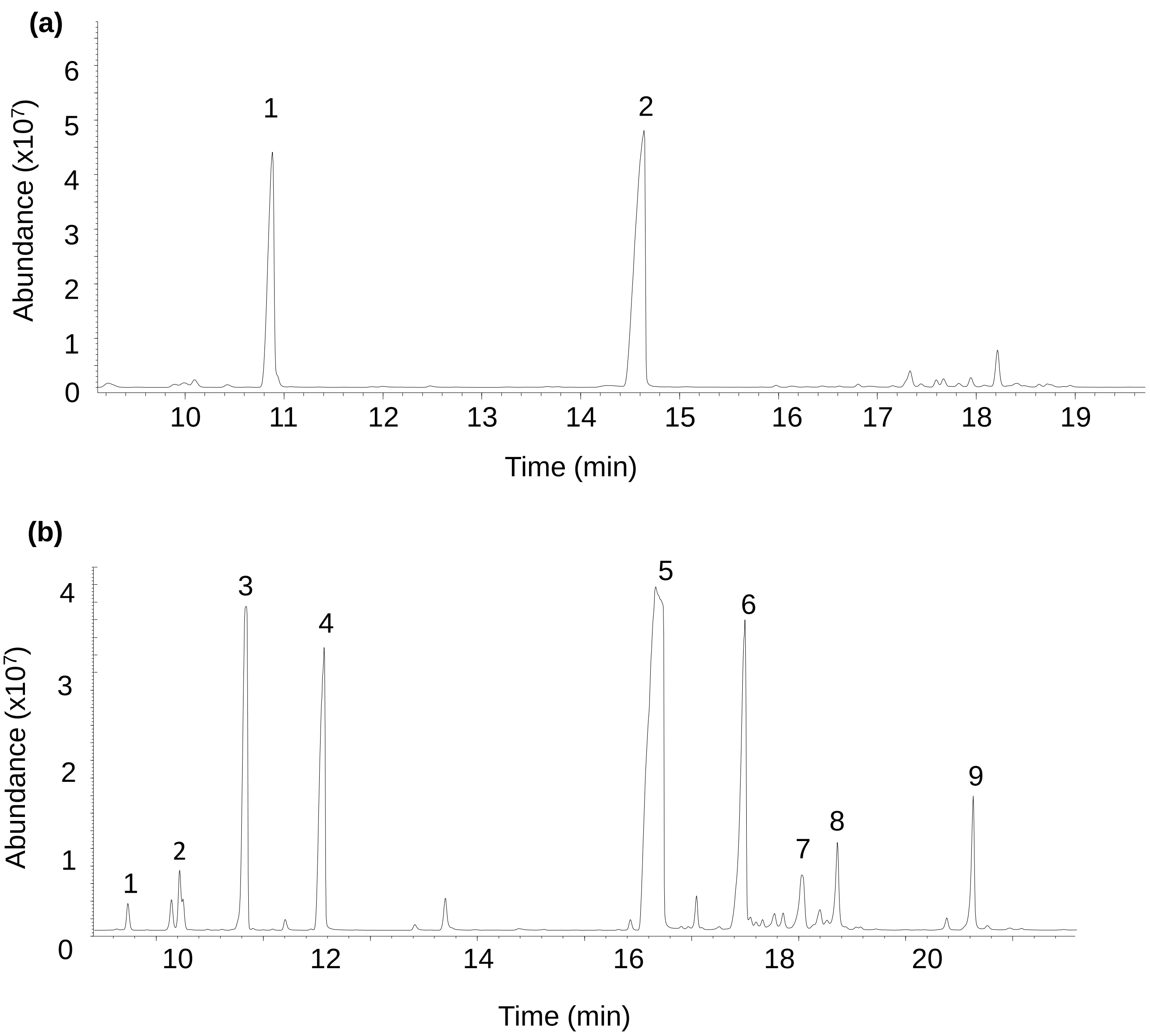

Figure S3. GC profiles of wild type plants. (a) Spearmint, 1. limonene; 2. carvone. (b) Sweet basil, 1. alpha-pinene; 2. beta-pinene; 3. eucalyptol; 4. linalyl acetate; 5. eugenol; 6. alpha-bergamotene; 7. germacrene D; 8.gamma-muurolene; 9. β-copaene.

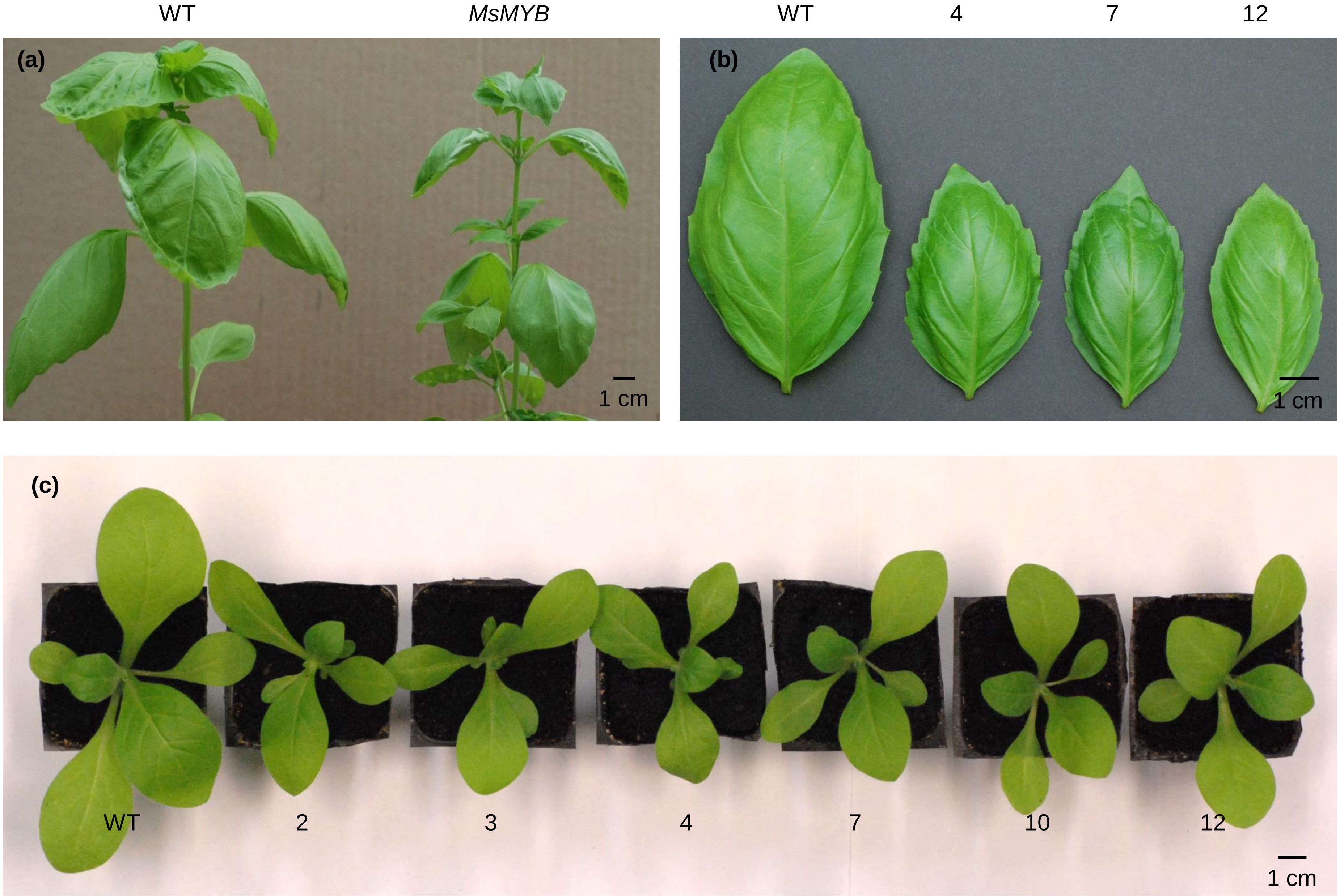

Figure S4. Transgenic plants overexpressing *MsMYB* show smaller leaf size. Ectopic expression of *MsMYB* led to decreased leaf size in transgenic basil plants (a), (b) and tobacco plants (c).

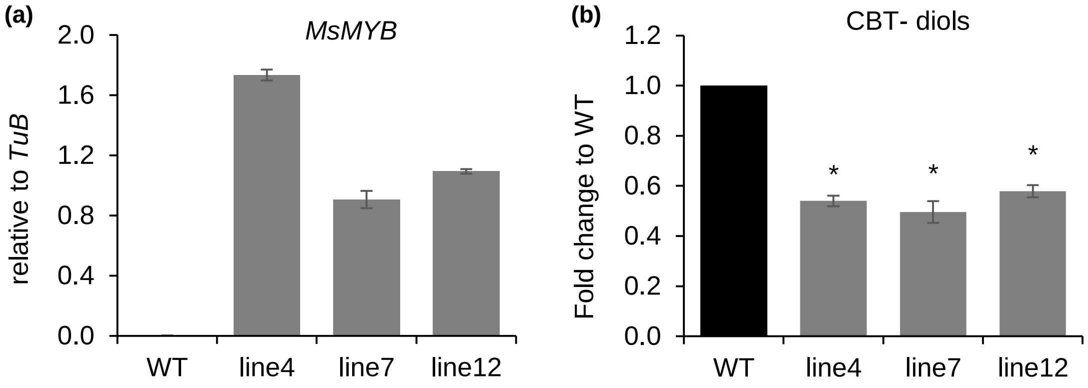

Figure S5. Ectopic expression of *MsMYB* in tobacco. (a) *MsMYB* expression in tobacco transgenic plants. (b) Reduced levels of CBT-diols in transgenic tobacco plants expressing *MsMYB.* Data are indicated as mean ± SE. *, $p < 0.05$; **, $p < 0.01$; ***, $p < 0.001$.

Supplemental Tables

Table S1. Primers used in this study.

Name	Sequence (5' to 3')	Purpose
MYB_5'_GSP1	TGATTTCATTGTCTGTTCTACCCG	RACE
MYB_5'_GSP2	TCAACCCAACTTTCTCACAGCAC	RACE
MYB_3'_GSP1	GCTAGATAACTCGTCGTGGCAAA	RACE
MYB_q_F	GGTTTCCGCCTCCCTAATCC	qPCR
MYB_q_R	CGACTCTTTCTCCGGAGTGG	qPCR
MYB_F	CACCATGGGAAGAGCGCCGTGCT	Subcellular localization
MYB_R	CAACAACCAAGAAAGCATTGCAC	Subcellular localization
MYB_OE_F	CACCATGGGAAGAGCGCCGTGCT	Overexpression
MYB_OE_R	TGACAACAACCAAGAAAGCATTGCAC	Overexpression
MYB_SphI	CGCATGCACTGAGATGGATTAATTAT	RNAi
MYB_HindIII	CAAGCTTCCTCGTCGTCCCAAATCCAC	RNAi
MYB_ XbaI	CTCTAGAGCACTTGCCGGGTAGAACAG	RNAi
MYB_ XhoI	GCTCGAGCCTCGTCGTCCCAAATCCAC	RNAi
MYB_GW_GPS1	GCATTCTTGGGCAATGATCGCCAGCAGCC	Genome walking
MYB_GW_GPS2	CAACTTTCTCACAGCACGGCGCTCTTCCC	Genome walking
35S(591)-F	CTCAGAAGACCAAAGGGCTATT	Probe for southern blot
35S(-34)-R	TGTTTGTTTTGTTGTGGTATTG	Probe for southern blot
GPS_LSU_q_F	GCAGGCCGACGAACCACAAGGT	qPCR
GPS_LSU_q_R	CGAGCAGATGTCCACCACCTGCC	qPCR
GPS_F	CACCATGAGTGTTCTTGTTAATCCTGTG	Subcellular localization
GPS_R	ATTGTCTCTATAAGCAATGTAATTGGCG	Subcellular localization
GPS_LSU_GW_GPS1	ATCTGGATCTCCGCCTCCGGCCGCCGT	Genome walking
GPS_LSU_GW_GPS2	CAACCCAGCACTGCAACTGGAAATCTGTGCG	Genome walking
EF1-F	TACTGCACTGTGATTGATGCC	qPCR
EF1-R	CATCCATCTTGTTACAGCAGC	qPCR

ObEF- F	AATGGCAAAAAGCTCGAAGA	qPCR
ObEF- R	TCGCAGACATGACAGACACA	qPCR
B_GPS_L_F	CTGCGAGCTGGTTGGCGGCG	qPCR
B_GPS_L_R	GGCCACGTGTTCGAACGCGAACGA	qPCR
NS_EF_F	AGGTACTGTGGCGACGGGGAGAGT	qPCR
NS_EF_R	GTGTGCGGAGTAATTGTTCCGGGC	qPCR
GFP_F	CACCATGGTGAGCAAGGGCGA	GUS assay
GFP_R	TTACTTGTACAGCTCGTCCATGCCG	GUS assay
P_MYB12_F	CACCAAATCATGTCGCCGTGTAG	Promoter expression
P_MYB12_R	ACCTAATGGAGTACTACTTATAGAGAC	Promoter expression
P_MsGPS_F	CACCATCATGGTTAAACATATGAA	Promoter expression
P_MsGPS_R	TTTTACCAACAGAAATATATATATATATAT	Promoter expression
ath-miR396a	UUCCACAGCUUUCUUGAACUG	miRNA qPCR
ms-miR858	UUCGUUGUCUGUUCGACCUUG	miRNA qPCR

Table S2. List of genes analyzed in spearmint transgenic plants.

**Enzymes**	Chavicol O-methyltransferase	flavonoid 3'-O-methyltransferase
DXS (1-deoxy-D-xylulose-5-phosphate (DXP) synthase)	Enolase	G6PD (glucose-6-phosphate dehydrogenase)
DXR (DXP reductoisomerase)	DAHPS (3-deoxy-d-arabino-heptulosonate 7-phosphate synthase)	Gibberellin 3-beta-dioxygenase
MCT (MEP cytidyltransferase)	FOMT (Tricetin 3',4',5'-O-trimethyltransferase)	Transketolase
CMK (4-(cytidine 5-diphospho)-2-C-methyl-D-erythritol kinase)	Aldolase	Sucrose synthase

MCS (2-C-methyl-D-erythritol 2,4-cyclodiphosphate (ME-2,4cPP) synthase)	SAM (methionine adenosyltransferase 3, S-adenosylmethionine synthetase)	Cytosolic invertase
HDS (1-hydroxy-2-methyl-2-butenyl 4-diphosphate (HMBPP) synthase)	FHY3 (far-red elongated hypocotyl 5)	**Transcription factors**
HDDR (HMBPP reductase)	Phospholipase A2	MYB112
IPPI (Isopentenyl diphosphate (IPP,C5) Delta-isomerase	ATPase	MYB4
LS (Limonene synthase)	Thioredoxin reductases	YABBY
L6OH (limonene-6-hydroxylase)	Cytochrome oxidase	**Transporters**
GPS (geranyl pyrophosphate synthase)	2-oxoglutarate (2OG) and Fe(II)-dependent oxygenase	Nonmitochondrial ATP/ADP Transporters
GGPS (geranylgeranyl pyrophosphate synthase)	Caffeic acid O-methyltransferase	Glucose 6-phosphate/phosphate translocator
FPS (farnesyl pyrophosphate synthase)	Phenylalanine ammonia lyase	ABC (ATP-binding cassette transporters)
NDS (neryl diphosphate synthase)	Chalcone isomerase	
Eugenol synthase	Cinnamate-4-hydroxylase	

Table S3. Oligonucleotides used for the generation of bait sequences.

S.No	*cis*-element repeat	Forward primer sequence	Reverse primer sequence
a	MYB binding site 1	AGCTTCAACCCAGCACTG**CAACTG**GAAATCTGTGCGAAGC	TCGAGCTTCGCACAGATTTC**CAGTTG**CAGTGCTGGGTTGA
b	MYB binding site 2	AGCTTTTGGCAGTTTAACGCC**TAACTG**CTTTTAAAGCCCTAAAC	TCGAGTTTAGGGCTTTAAAAG**CAGTTA**GGCGTTAAACTGCCAAAA
c	MYB binding site (Full GPS promoter)	AAGCTTATCATGGTTAAACATATGAAAAAAT	CTCGAGCAACCCAGCACTGCAACTGGAAATC
d	Mutant MYB binding site 1	AGCTTCAACCCAGCACTG**CAGGGG**GAAATCTGTGCGAAGC	TCGAGCTTCGCACAGATTTC**CCCCTG**CAGTGCTGGGTTGA
e	Mutant MYB binding site 2	AGCTTTTGGCAGTTTAACGCC**TAGGGG**CTTTTAAAGCCCTAAAC	TCGAGTTTAGGGCTTTAAAAG**CCCCTA**GGCGTTAAACTGCCAAAA

cis-element repeats are shown in bold and restriction sites have been underlined

Agrobacterium transformation of spearmint

Young leaves from in-vitro plants were the source of explants for transformation. They were submerged in agrobacterium culture (EHA105) and incubated at room temperature for 30 min with gentle shaking followed by vacuum infiltration for 5 min. The explants were air dried for 10 min and placed in cocultivation (CC) media plates (MS salts + sucrose (30 g/l) + BA (5 mg/l) + IBA (0.4 mg/l) + acetosyringone (200 µm/l)) for 3 days in dark. After cocultivation period the explants were washed with sterile water, dried and placed in shoot induction media plates (MS salts + sucrose (30 g/l) + BA (5 mg/l) + NAA (0.02 mg/l) + cefotaxime (150 mg/l) + kanamycin (30 mg/l)) in dark. After 4-5 weeks GFP positive shoots were selected and transferred to light. The well grown shoots were later transferred to basal media plates (MS salts + sucrose (30 g/l) + cefotaxime (100 mg/l) + kanamycin (50 mg/l)) for root induction. Plantlets with well-developed roots were transferred to soil and grown under greenhouse conditions.
